# Optimization of parathyroid ^11^C-choline PET protocol for localization of parathyroid adenomas in patients with primary hyperparathyroidism

**DOI:** 10.1186/s13550-019-0534-5

**Published:** 2019-07-31

**Authors:** Milou E. Noltes, Schelto Kruijff, Walter Noordzij, Eef D. Telenga, David Vállez García, Malgorzata Trofimiuk-Müldner, Marta Opalińska, Alicja Hubalewska-Dydejczyk, Gert Luurtsema, Rudi A. J. O. Dierckx, Mostafa El Moumni, Ronald Boellaard, Adrienne H. Brouwers

**Affiliations:** 1 Department of Surgery, University of Groningen, University Medical Center Groningen, Groningen, The Netherlands; 2 University of Groningen, University Medical Center Groningen, Department of Nuclear Medicine and Molecular Imaging, P.O. Box 30 001, 9700 RB Groningen, The Netherlands; 3 Department of Nuclear Medicine, Isala Hospital, Zwolle, The Netherlands; 4 Department of Endocrinology, Jagiellonian University, Medical College, Krakow, Poland; 5 Nuclear Medicine Unit, Department of Endocrinology, University Hospital, Krakow, Poland

**Keywords:** Primary hyperparathyroidism, ^11^C-Choline PET, Inter-observer agreement, Scan protocol

## Abstract

**Purpose:**

To evaluate the optimal tracer uptake time, the minimal amount of radioactivity and the inter-observer agreement for ^11^C-choline positron emission tomography/computed tomography (PET/CT) in patients with primary hyperparathyroidism (pHPT).

**Methods:**

Twenty-one patients with biochemically proven pHPT were retrospectively studied after injection of 6.3 ± 1.2 MBq/kg ^11^C-choline. PET data of the first nine patients, scanned for up to 60 min, were reconstructed in 10-min frames from 10- to 60-min postinjection (p.i.), mimicking varying ^11^C-choline uptake times. Parathyroid adenoma to background contrast ratios were calculated and compared, using standardized uptake values (SUVs). Data was reconstructed with varying scan durations (1, 2.5, 5, and 10 min) at 20–30-min p.i. (established optimal uptake time), mimicking less administered radioactivity. To establish the minimal required radioactivity, the SUVs in the shorter scan durations (1, 2.5, and 5 min) were compared to the 10-min scan duration to determine whether increased variability and/or statistical differences were observed. Four observers analyzed the ^11^C-choline PET/CT in four randomized rounds for all patients.

**Results:**

SUVpeak of the adenoma decreased from 30 to 40 p.i. onwards. All adenoma/background contrast ratios did not differ from 20- to 30-min p.i. onwards. The SUVs of adenoma in the scan duration of 1, 2.5, and 5 min all differed significantly from the same SUV in the 10-min scan duration (all *p* = 0.012). However, the difference in absolute SUV adenoma values was well below 10% and therefore not considered clinically significant. The inter-observer analysis showed that the Fleiss’ kappa of the 1-min scan were classified as “moderate,” while these values were classified as “good” in the 2.5-, 5-, and 10-min scan duration. Observers scored lower certainty scores in the 1- and 2.5-min scans compared to the 5- and 10-min scan durations.

**Conclusion:**

The optimal time to start PET/CT scanning in patients with pHPT is 20 min after mean injection of 6.3 MBq/kg ^11^C-choline, with a recommended scan duration of at least 5 min. Alternatively, the radioactivity dose can be lowered by 50% while keeping a 10-min scan duration without losing the accuracy of ^11^C-choline PET/CT interpretation.

**Electronic supplementary material:**

The online version of this article (10.1186/s13550-019-0534-5) contains supplementary material, which is available to authorized users.

## Background

Primary hyperparathyroidism (pHPT) is a common endocrine disorder, with the highest incidence in elderly females (> 70 years) [1]*.* Eighty to 90% of pHPT is caused by a single parathyroid adenoma [2]. Surgery, preferably a unilateral minimally invasive parathyroidectomy (MIP), is the only curative treatment.

To perform a MIP successfully, accurate preoperative imaging is essential. Worldwide, the current primary preoperative localization imaging standard consists of cervical ultrasonography (cUS) combined with ^99m^Tc-methoxyisobutylisonitrile-single-photon emission computed tomography/(computed tomography) *(*MIBI-SPECT/(CT)) [3, 4] reaching a sensitivity of 80–95% [5–7]. For the remaining 5–20% of patients, a full neck exploration is still necessary.

Lately, new functional imaging techniques using positron emission tomography (PET)/CT, with, e.g., ^11^C-methionine [8, 9] as radiotracer, have been studied, but no ideal PET tracer for adenomas has emerged so far. Recently, ^11^C/^18^F-choline was reported for visualization of adenomas and favorable results have been published in literature [10–15]. The physiology behind the uptake of ^11^C/^18^F-choline is unknown, but may be based on the elevated concentration of phosphatidylcholine in parathyroid cells [16].

So far, only one study has investigated the clinical performance of ^11^C-choline PET/CT in patients with pHPT [13]. Because ^11^C-choline is a relatively new technique in patients with pHPT, the optimal uptake time of the tracer and minimal injected radioactivity needs to be further defined. To fulfill the need of routine clinical practice, consistency in the interpretation between different observers is essential.

Therefore, this study aimed to optimize the scan protocol, by assessing the optimal scanning time of ^11^C-choline in combination with the minimal amount of radioactivity needed for clinically acceptable image quality. Also, we studied the inter-observer agreement of ^11^C-choline PET/CT for the detection of parathyroid adenomas in patients with pHPT.

## Material and methods

### Study design and patients

This is a single-center retrospective cohort study of patients with biochemically proven pHPT who underwent preoperative localization of the suspected parathyroid adenoma using ^11^C-choline PET/CT in a tertiary referral hospital.

The medical charts of all patients who underwent ^11^C-choline PET/CT between April 2015 and February 2017 were reviewed. Patients eligible for inclusion were those ≥ 18 years, diagnosed with biochemically confirmed pHPT, and who underwent ^11^C-choline PET/CT for the localization of the suspected adenoma. In total, 23 patients underwent ^11^C-choline PET/CT for the localization of an adenoma. All patients had biochemically confirmed pHPT (calcium and PTH values above the upper limit of normal). However, one patient was known to have multiple endocrine neoplasia type I, and one patient was diagnosed with familial hypocalciuric hypercalcemia (FHH); these two patients were excluded from this analysis. Of the analyzed patients that were operated (67%), the ^11^C-choline PET/CT correctly identified the location of the adenoma in all cases. Of the analyzed patients that were not operated (33%), the ^11^C-choline PET/CT was positive in all but one patient. This patient was only included in the inter-observer analysis of this study and not in the analysis of the optimal uptake time of the tracer and the radioactivity to be administered (data not shown).

The medical charts were reviewed to determine the injected activity of ^11^C–choline in MBq, gender, age, length, weight, preoperative PTH, and corrected calcium (for calculation of corrected calcium refer to Additional file 1).

Data obtained from patient records were anonymously stored using study-specific patient codes in a password-protected database. The study was exempt for collection of informed consent after reviewing by the Medical Ethics Committee Groningen (registration number 2016/413).

### ^11^C-Choline PET/CT

^11^C-choline was produced on site as described in [17]. Further details on production, patient preparation, and PET/CT acquisition can be found in Additional file 2. In the first nine patients, PET/CT images were taken directly after injection of 7.0 ± 0.5 MBq/kg [range 6.1–7.4 MBq/kg] ^11^C-choline for up to 40 to 60 min postinjection (p.i.). After an interim analysis of the first nine patients had been performed to determine the most suitable uptake time of the tracer to reduce overall scan duration, all subsequent patients were scanned dynamically 20 min after the injection of mean 5.9 ± 1.4 MBq/kg [range 4.2–8.5 MBq/kg] for a duration of 10 min.

### ^11^C-Choline PET/CT analysis

Of the first nine patients, the images were reconstructed in time frames of 10 min (0–10 to 50–60-min p.i.) to determine the optimal uptake time of the tracer. Also, these images were reconstructed in scan durations of 5 min (20–25 and 25–30 min), 2.5 min (20–22.5 to 27.5–30 min), and 1 min (20–21 to 29–30 min) to assess image quality as a function of scan duration used as a surrogate for variation in administered radioactivity.

For each of these newly created images, accumulated activity of ^11^C-choline in the adenoma was (semi) quantitatively evaluated by placing an automatic volume of interest (VOI) with a threshold of 50% of the maximum tracer uptake in the lesion, using an in-house software (ACCURATE) [18]. In the surrounding normal background tissues (descending aorta, thyroid, shoulder muscle, and the first thoracic vertebrae (T1)), a spherical or cubical VOI, with a fixed size, was positioned. In the thyroid gland, the cubical VOI was positioned in an area on the contralateral side of the adenoma in healthy appearing thyroid tissue. VOI’s were copied and transferred to scans of all time frames through an automatic linking feature and manual correction if needed. In each of these newly created images, peak, mean, and maximum standardized uptake values corrected for body weight (SUV_peak_, SUV_mean_, _and_ SUV_max_) were obtained for adenoma and background organs: descending aorta, thyroid, shoulder muscle, and T1. SUV_max_ represents the maximum tracer uptake seen across all voxels within the volume of interest. SUV_peak_ represents the average uptake in a 1 mL spherical volume of interest positioned such to yield the highest value across all possible locations of the lesion (or organ volume of interest). SUV_mean_ is the mean SUV within the volume of interest. SUV_mean_ was used for analysis of the background tissues, because it provides the most accurate and precise SUV in case of regions with an almost uniform uptake, and SUV_peak_ was used for the adenoma being the preferred SUV metric to quantify tracer uptake in (small) lesions besides SUV_max_ [19]. Moreover, SUV_max_ was used for all as this metric is and has been used frequently and is therefore included to allow comparison with other studies.

### Inter-observer agreement

All patients were included in the inter-observer agreement analysis. The acquired image data at 20-min p.i. was post-processed with varying scan duration of 1, 2.5, 5, and 10 min, respectively.

From all patients, two scans with a scan duration of 1 min and 2.5 min, both scans with a duration of 5 min, and the scan with a duration of 10 min were randomly selected.

Four observers visually interpreted the scans in four rounds, with increasing scan duration per round. Observers were asked to identify and localize any abnormally increased ^11^C-choline uptake and to localize it in the right upper, left upper, right lower, left lower, or ectopic zones. Next, in case of a focal increased uptake, per location, they had to score how certain they were that the uptake was indeed increased (certainty of increased uptake (CIU)) and how certain they were that the uptake could be attributed to an adenoma (certainty of adenoma (CA)). They completed a standard scoring form per patient, per round including a 5-point scale to score their CIU and CA, with “1” being totally unsure and “5” being totally sure. Further details of the inter-observer analysis and assessment can be found in Additional file 3.

### Statistical analysis

Patient characteristics are described using means and standard deviations (SD) or medians and ranges for continuous variables (depending on normal distribution). All statistical analyses were performed with IBM SPSS Statistics for Windows, Version 23.0 (Armonk, NY: IBM Corp.). *P* values of < 0.05 were considered statistically significant. For multiple testing, the Bonferroni correction was applied. Graphics were generated using GraphPad Prism, Version 7.02 (La Jolla, CA, USA).

#### Optimal uptake time of tracer

Of the first nine patients, scanned for up to 40 to 60 min, contrast ratios (adenoma to muscle, T1, aorta, and thyroid tissue) were calculated to determine the optimal uptake time of the tracer. Contrast ratios were calculated by dividing the SUV_peak_ for the adenoma by the SUV_mean_ for the background. The ratios in every time frame were compared with the ratios in the upcoming time frame using the Wilcoxon signed-rank test as normality of the data could not be shown.

The SUV_mean_ for thyroid and SUV_peak_ for adenoma (of the first nine patients) in every uptake time were compared with the same SUVs in the upcoming uptake time using the Wilcoxon signed-rank test as normality of the data could not be shown. Using the Bonferroni correction, *p* values < 0.01 were considered statistically significant.

#### Radioactivity to be administered

In the analysis of the radioactivity to be administered, only the first eight patients (scanned for up to 40 to 60 min) were included, since no post-processed images could be made in scan durations of 1, 2.5, and 5 min for one patient, because the original raw data was no longer available. The post-processed images with a scan duration of 10 min was, however, still available.

To assess the effect of injected radioactivity on SUV_max_, SUV_mean_, and SUV_peak_ for adenoma and background tissues, the SUVs in the shorter scan durations (1, 2.5, and 5 min) were compared to the 10-min scan duration (at the determined optimal uptake time) to determine whether statistical differences were observed, using the Wilcoxon signed-rank test as normality of the data could not be shown. For these analyses, the average SUVs of patients per scan duration were calculated, since the 1-, 2.5-, and 5-min scan durations had multiple measurements of SUV (10, 4, and 2, respectively) and the 10-min scan duration only had 1 measurement. In this way, we could assess if using different scan durations would result in different SUV data, on average, i.e., if it would result in a systematic bias. Using the Bonferroni correction, *p* values < 0.017 were considered statistically significant.

#### Inter-observer agreement

Inter-observer agreement was calculated per possible location (right upper, left upper, right lower, left lower, ectopic) by comparing the results of the location of the adenoma using the Fleiss’ kappa. Interpretations of the kappa values were as follows: < 0.00 poor, 0.00–0.20 slight, 0.21–0.40 fair, 0.41–0.60 moderate, 0.61–0.80 good, and > 0.81 almost perfect agreement [20].

## Results

### Patient and scan characteristics

A total of 21 patients (15 females and 6 males) was included in this study, with a mean age of 62 years [range 36–83] and a mean administered activity of 6.3 ± 1.2 MBq/kg [range 4.2–8.5 MBq/kg] (Table 1).

**Table 1:** Patients’ baseline and ^11^C-choline PET/CT scan characteristics

Number	Gender	Age	Weight (kg)	Height (m)	PTH (pmol/L)	Corrected calcium (mmol/L)	Time to start acquisition	Duration ^11^C-choline PET/CT	Injected activity ^11^C-choline (MBq/kg)
1	F	68	75	1.68	9.3	2.54	Directly p.i.	60 min	7.2
2	F	51	81	1.78	8.9	2.81	Directly p.i.	60 min	6.1
3	F	52	87	1.69	7.9	2.60	Directly p.i.	40 min	7.3
4	F	47	65	1.65	17.5	2.73*	Directly p.i.	50 min	7.4
5	F	65	75	1.72	8.8	2.67	Directly p.i.	60 min	7.0
6	F	74	92	1.60	28.0	3.10	Directly p.i.	50 min	7.3
7	M	36	73	1.83	47.0	2.93	Directly p.i.	60 min	7.0
8	M	53	80	1.81	10.0	2.69	Directly p.i.	60 min	7.1
9	F	63	89	1.72	8.3	2.68	Directly p.i.	40 min	6.2
10	F	69	90	1.78	18.0	2.72	20-min p.i.	10 min	4.6
11	M	60	72	1.78	14.0	2.62	20-min p.i.	10 min	5.6
12	M	83	93	1.82	10.0	2.65*	20-min p.i.	10 min	4.3
13	F	71	64	1.69	15.0	2.56	20-min p.i.	10 min	6.7
14	F	72	52	1.61	11.3	2.94	20-min p.i.	10 min	8.5
15	F	60	79	1.79	9.0	2.79*	20-min p.i.	10 min	5.3
16	F	68	53	1.61	27.1	3.00	20-min p.i.	10 min	7.5
17	F	53	70	1.70	11.3	2.94*	20-min p.i.	10 min	5.6
18	F	63	67	1.75	9.1	2.61	20-min p.i.	10 min	6.6
19	F	63	67	1.64	16.4	2.62	20-min p.i.	10 min	6.9
20	M	68	90	1.70	10.3	2.73*	20-min p.i.	10 min	4.6
21	M	69	102	1.84	15.0	2.59	20-min p.i.	10 min	4.2

*F* female, *M* male, *p.i.* postinjection

*only non-corrected calcium level was available

### ^11^C-Choline PET/CT analysis

#### Optimal uptake time of tracer

**Fig. 1:**
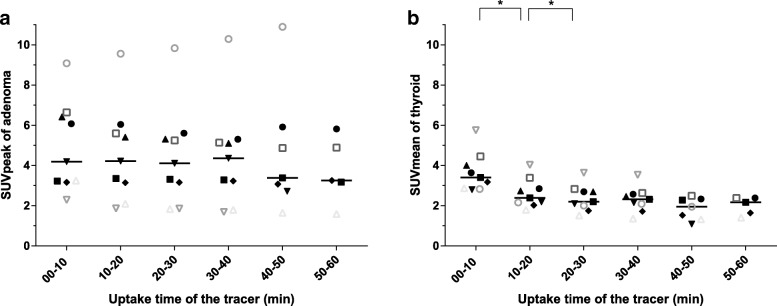
Scatter plot of SUVs for parathyroid adenoma and thyroid in *n* = 9 patients. Scatter plots with median values of **a** SUV_peak_ of the adenoma. **b** SUV_mean_ of the thyroid. *SUV* standardized uptake value, *Uptake time of tracer* time (in min) between injection of ^11^C-choline and start of PET/CT acquisition, *significant difference (*p* < 0.01). Only 7 and 5 patients were scanned until 50-min and 60-min postinjection, respectively

The SUV_mean_ for thyroid initially decreased before leveling off from 20- to 30-min p.i. onwards, while the SUV_peak_ for adenoma was constant until 30–40-min p.i. and decreased afterwards (Fig. 1) (Table 2). We found no significant differences between the SUV_peak_ for adenoma for the different uptake times (*p* > 0.110). There were significant differences between the SUV_mean_ for thyroid in the uptake time of 0–10 versus 10–20 min (*p* = 0.008) and that of 10–20 versus 20–30 min (*p* = 0.008). From 20- to 30-min p.i. onwards, there were no significant differences in the SUV_mean_ for thyroid (*p* > 0.018).

**Table 2:** Descriptive statistics of the SUVs for thyroid and adenoma for the different uptake times in *n* = 9 patients

	Uptake time of tracer (min)					
	00–10	10–20	20–30	30–40	40–50	50–60
SUV_mean_ thyroid						
Median	3.41	2.38	2.20	2.32	1.95	2.18
SD	0.97	0.72	0.65	0.62	0.55	0.45
IQ	1.02	1.21	1.13	1.06	0.99	0.86
SUV_peak_ adenoma						
Median	4.19	4.22	4.11	4.36	3.38	3.25
SD	2.24	2.39	2.46	2.58	3.10	1.64
IQ	3.16	3.19	2.93	2.70	3.03	2.97

*SD* standard deviation, *IQ* interquartile range, *SUV* standardized uptake value

Only 7 and 5 patients were scanned until 50 and 60 min, respectively

We focused on the adenoma/thyroid contrast ratio, as the adenoma is usually closest located to the thyroid, refer to Additional file 4 for data on adenoma/aorta, adenoma/muscle and adenoma/T1 contrast ratios.

**Fig. 2:**
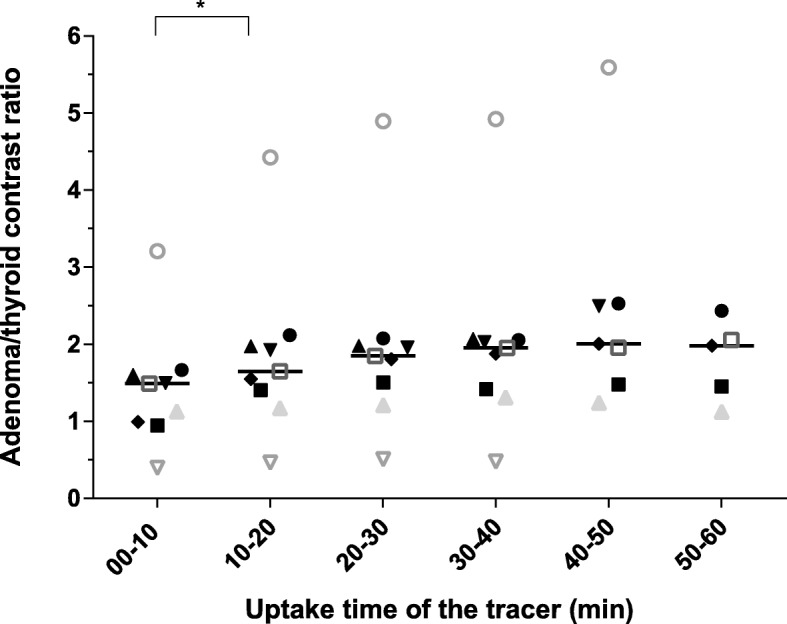
Scatter plot of parathyroid adenoma to thyroid contrast ratios in *n* = 9 patients. Scatter plot with median values of adenoma/thyroid ratio. *Uptake time of tracer* time (in min) between injection of ^11^C-choline and scanning procedure, *significant difference (*p* < 0.01). Only 7 and 5 patients were scanned until 50-min and 60-min postinjection, respectively

The adenoma/thyroid ratio became constant from the uptake time of 20–30-min p.i. onwards (Fig. 2). In the uptake time of 0–10 versus 10–20-min p.i., the adenoma/thyroid ratio increased from 1.49 to 1.65 (*p* = 0.008) (Table 3). In the uptake time of 10–20 versus 20–30 min, the adenoma/thyroid ratio (increase from 1.65 to 1.85) is just slightly beyond the level of significance (*p* = 0.028). From the uptake time of 20–30-min p.i. onwards, there were no significant differences with upcoming uptake times for any ratio and *p* values were higher compared to previous uptake times (*p* > 0.043).

**Table 3:** Descriptive statistics of the adenoma/thyroid ratios for the different uptake times in *n* = 9 patients

	Uptake time of tracer (min)					
	00–10	10–20	20–30	30–40	40–50	50–60
Median	1.49	1.65	1.85	1.95	2.01	1.98
SD	0.78	1.09	1.20	1.21	1.46	.52
IQ	0.61	0.59	0.61	0.64	0.91	0.95

*SD* standard deviation, *IQ* interquartile range

Only 7 and 5 patients were scanned until 50 and 60 min, respectively

#### Radioactivity to be administered

The SD and interquartile range (IQ) of SUV_peak_ for adenoma, SUV_mean_ for background tissue, and SUV_max_ for all locations decreased with increasing scan duration (Table 4 and Additional file 5). The SUV_peak_ for adenoma and SUV_mean_ for background tissue varied less than SUV_max,_ as witnessed by higher SD and IQ for SUV_max_ for each scan duration (Table 4 and Additional file 5).

**Table 4:** Descriptive statistics of the average SUVs for adenoma and thyroid for the different scan durations in *n* = 8 patients

SUV_peak_ for adenoma					SUV_mean_ for thyroid				
Scan duration (min)					Scan duration (min)				
	1	2.5	5	10		1	2.5	5	10
Median	3.88	3.81	3.80	3.71	Median	2.10	2.11	2.11	2.15
SD	2.70	2.69	2.67	2.61	SD	0.69	0.69	0.69	0.68
IQ	3.41	3.43	3.44	3.34	IQ	0.96	0.97	0.96	0.99
SUV_max_ for adenoma					SUV_max_ for thyroid				
Scan duration (min)					Scan duration (min)				
	1	2.5	5	10		1	2.5	5	10
Median	6.74	6.47	6.41	6.24	Median	3.11	2.93	2.87	2.84
SD	4.06	3.98	3.89	3.71	SD	0.94	0.97	0.98	0.96
IQ	6.54	6.54	6.58	5.85	IQ	1.62	1.59	1.56	1.54

*SD* standard deviation, *IQ* interquartile range, *SUV* standardized uptake value

**Fig. 3:**
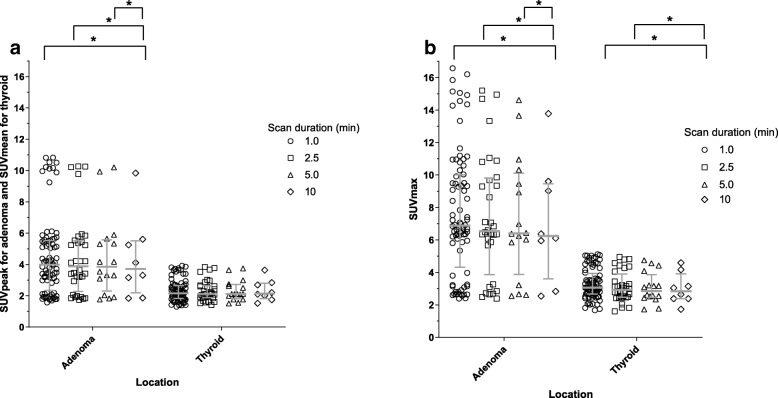
Scatter plot of SUV_mean_, SUV_peak_, and SUV_max_ of adenoma and thyroid in the different scan durations (1, 2.5, 5, and 10 min) in *n* = 8 patients. Scatter plot representing median values and interquartile ranges of **a** SUV_peak_ for adenoma and SUV_mean_ for thyroid in the different scan durations. **b** SUV_max_ for adenoma and thyroid in the different scan durations. *SUV* standardized uptake value, *significant difference (*p* < 0.017)

In addition, all individual SUVs in the scans with a duration of 1 min seemed more variable than the SUVs in the scan duration of 2.5, 5, and 10 min (Fig. 3, Table 4, and Additional file 5).

There was no significant difference in SUV_mean_ for thyroid between the different scan durations (all *p* = 0.674), whereas there was a significant difference in SUV_max_ for thyroid between a 1-min versus 10-min scan duration and a 2.5-min versus 10-min scan duration (both *p* = 0.012). There was no difference in SUV_max_ for thyroid between a 5-min versus 10-min scan duration (*p* = 0.069). The SUVs for adenoma in the scan duration of 1, 2.5, and 5 min all differed statistically from the same SUV in the 10-min scan duration (all *p* = 0.012).

#### Inter-observer agreement

**Fig. 4:**
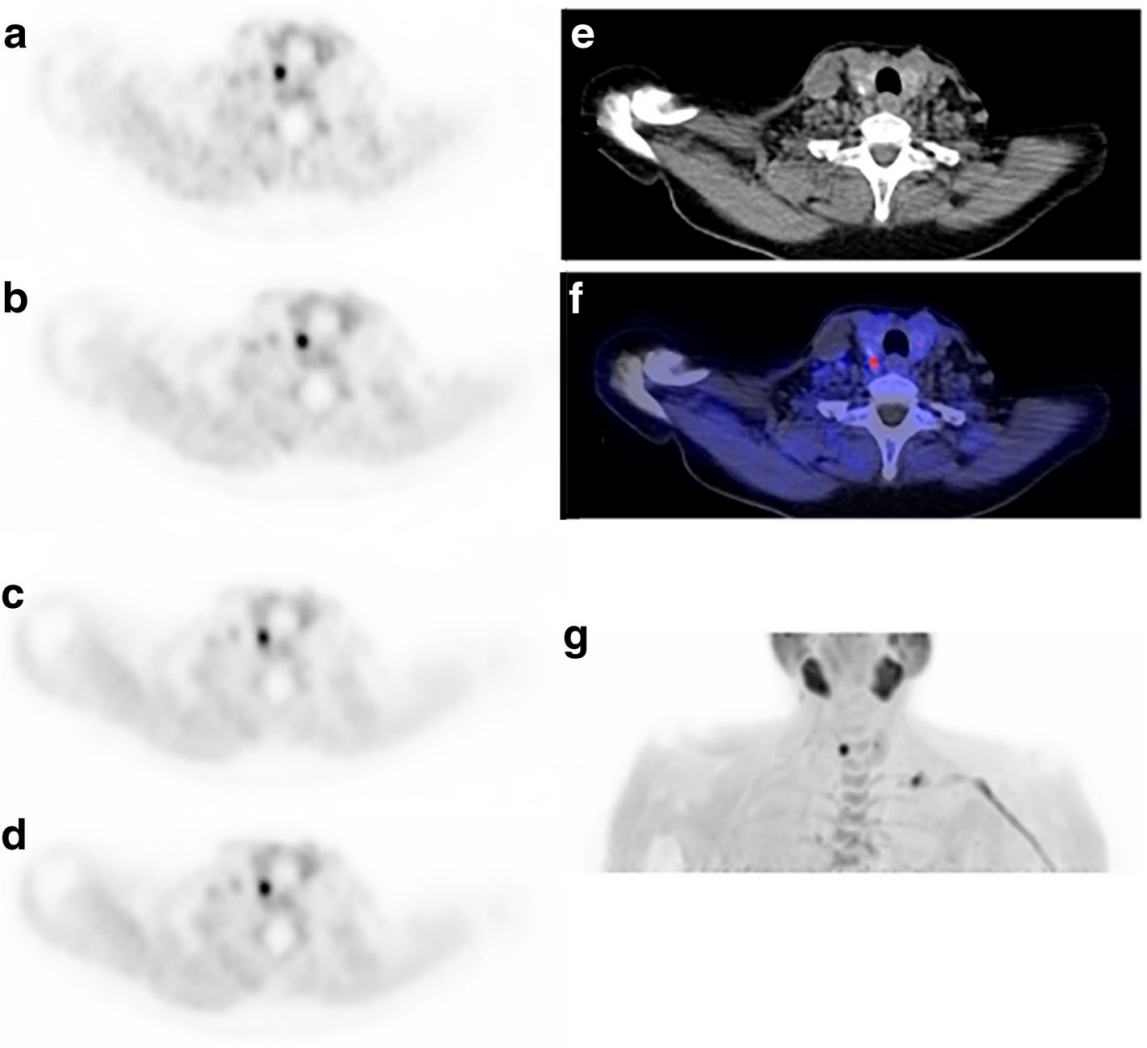
^11^C-Choline PET/CT images of a patient with a parathyroid adenoma 20-min p.i. with varying acquisition times. **a** Transverse PET image of 1-min acquisition, **b** 2.5-min acquisition, **c** 5-min acquisition, and **d** 10-min acquisition. **a** and **b** appear “noisier” than **c** and **d**. **e** Corresponding transverse image of low-dose CT, and **f** PET/CT fusion image with 5-min PET acquisition time. **g** Maximum Intensity Projection (MIP) PET image of 5-min acquisition at 20-min postinjection images of patient number 1 (Table 1). Note the physiological uptake in the salivary glands, and remaining activity in the vessel used for injection of the tracer. Also, slight uptake is seen in a thyroid nodule in the left thyroid lobe

In total, 141 scans were used for this analysis. Figure 4 shows a typical example of a ^11^C-choline PET/CT scan with 1-, 2.5-, 5-, and 10-min scan duration. The kappa values in the scan durations of 2.5, 5, and 10 min were good, while it was “moderate” in the scan duration of 1 min (Table 5). Most certainty scores, only representing the certainty score of positive identified lesions, increased with increasing scan duration (Table 6).

**Table 5:** Results of inter-observer agreement of *n* = 4 observers for the different scan durations in *n* = 21 patients

	1 min	2.5 min	5 min	10 min
Fleiss’ kappa location	0.553	0.687	0.656	0.674

**Table 6:** Descriptive statistics of the CIU and CA per observer (*n* = 4), only representing the certainty score of positive identified lesions, for the different scan durations in *n* = 21 patients

Scan duration of 1 min					Scan duration of 2.5 min				
	Obs1	Obs2	Obs3	Obs4		Obs1	Obs2	Obs3	Obs4
Mean CIU	3.54	2.56	4.55	4.40	Mean CIU	4.09	2.89	4.51	4.18
SD CIU	1.36	0.97	0.67	1.06	SD CIU	0.97	1.09	0.71	0.97
Range CIU	4	3	2	4	Range CIU	4	4	2	3
Mean CA	3.42	2.22	3.74	3.60	Mean CA	3.30	2.59	4.27	3.85
SD CA	1.30	1.01	1.36	1.24	SD CA	1.40	1.08	1.15	1.35
Range CA	4	3	4	4	Range CA	4	4	4	4
Scan duration of 5 min					Scan duration of 10 min				
	Obs1	Obs2	Obs3	Obs4		Obs1	Obs2	Obs3	Obs4
Mean CIU	4.21	4.00	4.77	3.94	Mean CIU	4.40	4.19	4.41	3.95
SD CIU	1.02	0.96	0.49	1.39	SD CIU	0.91	0.91	0.85	1.50
Range CIU	3	3	2	4	Range CIU	3	3	2	4
Mean CA	3.92	3.44	4.26	3.29	Mean CA	4.00	3.69	4.27	3.10
SD CA	1.25	1.01	1.31	1.47	SD CA	1.25	0.95	0.88	1.65
Range CA	4	3	4	4	Range CA	4	3	2	4

*CIU* certainty increased uptake, *CA* certainty adenoma, *Obs* observer, *SD* standard deviation

## Discussion

In this study, we evaluated the scan protocol and inter-observer agreement of ^11^C-choline PET/CT in patients with biochemically confirmed pHPT. We found that the optimal uptake time of ^11^C-choline PET/CT scanning is 20 min, since from 20-min p.i. onwards the adenoma/background contrast ratios and SUV_mean_ for thyroid became constant.

In addition, we quantitatively analyzed various scan durations as a surrogate for injecting different amounts of radioactivity. Although there was a significant difference in (average) SUVs at shorter scan durations compared to the 10-min scan, the differences for the 2.5- and 5-min scan durations were small, well below 10%, and clinically not relevant given the spread in the observed SUV values. However, the observed increased spread in SUV values for the 1-min scan duration was wider compared with the other scan durations. Therefore, we advise for quantitative analysis not to lower the scan duration beyond 2.5 min.

To evaluate the clinical relevance of images with different quality, we performed an inter-observer study regarding lesion detection and localization. We concluded that a 1-min scan is too short for accurate visual interpretation of ^11^C-choline PET/CT images. The certainty scores (CIU and CA) were generally lower in the 1-min and 2.5-min scan durations compared to the 5- and 10-min scan durations. In clinical routine, it may occur that the expected dose of ^11^C-choline is not fully administered to the patient, e.g., due to a lower production at the radiochemistry lab or to paravasal injection. Also, adenomas can be very small structures. Therefore, combining the results of the quantitative and inter-observer data, we recommend to establish a safety margin for ^11^C-choline PET/CT scanning with 6.3 MBq/kg and scan for at least 5 min. Alternatively, the radioactivity dose can be lowered by 50% while maintaining a 10-min scan duration.

As can be expected, we found that the SUV_peak_ is less variable than the SUV_max_. This is consistent with findings for FDG PET/CT in oncology, as shown by Lodge et al. and Makris et al. [21, 22]. In these studies, it was observed that SUV_max_ is more sensitive to noise, showing increased variability and upward bias as compared to SUV_peak_. The latter can be understood as SUV_max_ represents the uptake in a single voxel, while SUV_peak_ is derived from a 1 mL spherical volume of interest thereby mitigating the effects of image noise. Therefore, if the radioactivity in the adenoma needs to be quantified, it is best to use the SUV_peak_ for parathyroid tracer uptake quantification as it shows a better precision than SUV_max_.

SUVs in the blood pool are known to be relatively low, and thus, these regions suffer from poor count statistics. SUV_max_ for the aorta in the 1-min scan duration was higher than those seen at longer scan durations. This can be expected because SUV_max_ is more sensitive to noise and thus showing upward biases with increased noise levels [21, 22].

Quantitative research on the scan protocol for parathyroid imaging with PET/CT is very limited. The optimal scan time for ^18^F-choline PET/CT in pHPT has been investigated once before by Rep et al. ^18^F has a considerable longer half-life of 110 min, compared to 20 min for ^11^C. Rep et al. only focused on an uptake time of 5 min, 60 min, or 2 h. They found that the optimal scan time of ^18^F-choline PET/CT for localization of enlarged parathyroid tissue is 1 h after administration [23]. In the current study with ^11^C-choline, we focused on the interval between 5 and 60 min. Prabhu et al. also investigated the utility of an early dynamic PET/CT in detecting parathyroid lesions, but they performed it for the tracer ^18^F-choline. They found that early dynamic ^18^F-choline PET/CT imaging could suffice, without the need for a delayed image after 45 min [24]. This is more in line with the optimal uptake time of 20 min found in our study for ^11^C-choline.

Our retrospective study has limitations. We assumed that all lesions that were identified on the ^11^C-choline PET/CT were parathyroid adenomas in this group of patients with biochemically proven pHPT, although we only have surgical and pathological confirmation in two thirds of the patients (data not shown). Since our study focuses on the technical aspects of lesion detectability, and because determining the sensitivity of this type of PET/CT scan for correct localization of an adenoma was not the aim of our analysis, we believe for our study objective final surgical and pathological outcome is less crucial.

In our retrospective study, we chose to use SUV corrected for body weight, as this is the SUV type most used. No efforts were made to relate optimal dose to length, weight, or BMI. The latter would require a pharmacokinetic study including arterial sampling to assess which SUV normalization corresponds best with full quantitative pharmacokinetic results. This was beyond the scope of this study, and therefore, the most standard metric was chosen of SUV corrected for body weight. Yet, when analyzing the data using lean body mass (data not shown), results were very comparable and did not affect conclusions. Moreover, the sample size is too small to fully assess the relationship and the interactions between these parameters. Larger datasets are required for further refinement or dose optimization. Yet, at present, our study demonstrated that we can safely reduce scan duration or activity dose with a factor of two without compromising visual image interpretations. The authors acknowledge, however, that investigation of further dose refinements is of interest.

Always the same researcher quantified the ^11^C-choline PET/CT scans by placing the VOIs in the adenoma and background. Another researcher would probably find slightly different SUVs. To better estimate this standard error, we tested whether the SUV_mean_ was different if we moved the square VOI of the thyroid 2 voxels up or down. In the 10-min scan durations, the median difference was 3.32% with a standard deviation of 8.33%, which we consider acceptable low (data not shown).

Other limitations were encountered in the inter-observer part of the study. The working experience for the observers differed, and two of the observers had limited experience with ^11^C-choline PET/CT for the identification of adenomas. Despite these limitations, the inter-observer agreement for the scan duration of 2.5, 5, and 10 min qualified as “good.”

## Conclusion

This study optimizes the protocol for parathyroid ^11^C-choline PET/CT imaging, potentially resulting in less radioactivity injected into patients. We showed that, taking into account both quantitative performance and image quality, the optimal time to start PET/CT scanning in patients with pHPT is 20 min after mean injection of 6.3 MBq/kg ^11^C-choline, with a recommended scan duration of at least 5 min. Alternatively, the radioactivity dose can be lowered by 50% while maintaining a 10-min scan duration without losing accuracy of ^11^C-choline PET/CT interpretation.

## Additional files

Additional file 1: Corrected calcium (PDF 152 kb)

Additional file 2: ^11^C-choline PET/CT (DOCX 16 kb)

Additional file 3: Inter-observer agreement (DOCX 15 kb)

Additional file 4: Results optimal uptake time of tracer (DOCX 125 kb)

Additional file 5: Results radioactivity to be administered (DOCX 196 kb)

## Abbreviations

CA: Certainty adenoma
CIU: Certainty increased uptake
CT: Computed tomography;
cUS: Cervical ultrasonography
FHH: Familial hypocalciuric hypercalcemia
IQ: Interquartile range
MIBI-SPECT: ^99m^Tc-Methoxyisobutylisonitrile-single-photon emission computed tomography
MIP: Minimally invasive parathyroidectomy
p.i.: Postinjection
PET: Positron emission tomography
pHPT: Primary hyperparathyroidism
SD: Standard deviation
SUV: Standardized uptake value
T1: Thoracic vertebra T1
VOI: Volume of interest

## References

[1] Yeh MW, Ituarte PH, Zhou HC (2013). Incidence and prevalence of primary hyperparathyroidism in a racially mixed population. J Clin Endocrinol Metab.

[2] Clark OH, Duh Q, Kebebew E (2005). Textbook of endocrine surgery.

[3] Bilezikian JP, Khan AA, Potts JT (2009). Guidelines for the management of asymptomatic primary hyperparathyroidism. J Clin Endocrinol Metab.

[4] Hindié E, Ugur O, Fuster D (2009). 2009 EANM parathyroid guidelines. Eur J Nucl Med Mol Imaging.

[5] Sukan A, Reyhan M, Aydin M (2008). Preoperative evaluation of hyperparathyroidism: the role of dual-phase parathyroid scintigraphy and ultrasound imaging. Ann Nucl Med.

[6] Akbaba G, Berker D, Isik S (2012). A comparative study of pre-operative imaging methods in patients with primary hyperparathyroidism: ultrasonography, 99mTc sestamibi, single photon emission computed tomography, and magnetic resonance imaging. J Endocrinol Investig.

[7] Patel CN, Salahudeen HM, Lansdown M, Scarsbrook AF (2010). Clinical utility of ultrasound and 99mTc sestamibi SPECT/CT for preoperative localization of parathyroid adenoma in patients with primary hyperparathyroidism. Clin Radiol.

[8] Noltes ME, Coester AM, van der Horst-Schrivers AN (2017). Localization of parathyroid adenomas using 11C-methionine pet after prior inconclusive imaging. Langenbecks Arch Surg.

[9] Yuan L, Liu J, Kan Y, Yang J, Wang X (2017). The diagnostic value of 11C-methionine PET in hyperparathyroidism with negative 99mTc-MIBISPECT: a meta-analysis. Acta Radiol.

[10] Parvinian A, Martin-Macintosh EL, Goenka AH, Durski JM, Mullan BP, Kemp BJ, Johnson GB (2018). ^11^C-Choline PET/CT for detection and localization of parathyroid adenomas. AJR Am J Roentgenol.

[11] Treglia Giorgio, Piccardo Arnoldo, Imperiale Alessio, Strobel Klaus, Kaufmann Philipp A., Prior John O., Giovanella Luca (2018). Diagnostic performance of choline PET for detection of hyperfunctioning parathyroid glands in hyperparathyroidism: a systematic review and meta-analysis. European Journal of Nuclear Medicine and Molecular Imaging.

[12] Quak E, Blanchard D, Houdu B (2018). F18-choline PET/CT guided surgery in primary hyperparathyroidism when ultrasound and MIBI SPECT/CT are negative or inconclusive: the APACH1 study. Eur J Nucl Med Mol Imaging.

[13] Orevi M, Freedman N, Mishani E, Bocher M, Jacobson O, Krausz Y (2014). Localization of parathyroid adenoma by ^11^C-choline PET/CT: preliminary results. Clin Nucl Med.

[14] Broos Wouter A.M., van der Zant Friso M., Knol Remco J.J., Wondergem Maurits (2019). Choline PET/CT in parathyroid imaging. Nuclear Medicine Communications.

[15] Beheshti M, Hehenwarter L, Paymani Z (2018). 18F-Fluorocholine PET/CT in the assessment of primary hyperparathyroidism compared with 99mTc-MIBI or 99mTc-tetrofosmin SPECT/CT: a prospective dual-centre study in 100 patients. Eur J Nucl Med Mol Imaging.

[16] Hara T, Kosaka N, Kishi H (1998). PET imaging of prostate cancer using carbon-11-choline. J Nucl Med.

[17] Hessels-Scheper JG, Maarsingh P, Kwizera C (2014). GMP compliant radiosynthesis of11C and18F-labeled PET radiopharmaceuticals with a modular disposable cassette system. Eur J Nucl Med Mol Imaging.

[18] Boellaard R (2018). Quantitative oncology molecular analysis suite: ACCURATE. J Nucl Med.

[19] Boellaard R, Delgado-Bolton R, Oyen WJ (2015). FDG PET/CT: EANM procedure guidelines for tumour imaging: version 2.0. Eur J Nucl Med Mol Imaging.

[20] Landis JR, Koch GG (1977). The measurement of observer agreement for categorical data. Biometrics.

[21] Lodge MA, Chaudhry MA, Wahl RL (2012). Noise considerations for PET quantification using maximum and peak standardized uptake value. J Nucl Med.

[22] Makris NE, Huisman MC, Kinahan PE, Lammertsma AA, Boellaard R (2013). Evaluation of strategies towards harmonization of FDG PET/CT studies in multicentre trials: comparison of scanner validation phantoms and data analysis procedures. Eur J Nucl Med Mol Imaging.

[23] Rep S, Lezaic L, Kocjan T (2015). Optimal scan time for evaluation of parathyroid adenoma with [(18) F]-fluorocholine PET/CT. Radiol Oncol.

[24] Prabhu M, Kumari G, Damle NA (2018). Assessment of the role of early dynamic PET/CT with 18F-fluorocholine in detection of parathyroid lesions in patients with primary hyperparathyroidism. Nucl Med Commun.

