# Supplementary material for: Optimization of parathyroid 11C-choline PET protocol for localization of parathyroid adenomas in patients with primary hyperparathyroidism
Source: EJNMMI Res. 2019 Jul 31;9:73. doi: 10.1186/s13550-019-0534-5 (PMC6669228; doi:10.1186/s13550-019-0534-5)
Supplement: Supplementary file 1 — Corrected calcium (PDF 152 kb) [file 13550_2019_534_MOESM1_ESM.pdf]

# Optimization of parathyroid $^{11}$C-choline PET protocol for localization of parathyroid adenomas in patients with primary hyperparathyroidism

Milou E Noltes, Schelto Kruijff, Walter Noordzij, Eef D Telenga, David Vállez García, Malgorzata Trofimiuk-Müldner, Marta Opalińska, Alicja Hubalewska-Dydejczyk, Gert Luurtsema, Rudi AJO Dierckx, Mostafa El Moumni, Ronald Boellaard, Adrienne H Brouwers

**Correspondence to:**

A.H. Brouwers, MD, PhD

Department of Nuclear Medicine and Molecular Imaging

University Medical Center Groningen

a.h.brouwers@umcg.nl

## Additional file 1: Corrected calcium

Corrected calcium was calculated using the following formula: Ca + ((40 – Alb) × 0.02). ‘Ca’ is the serum calcium (mmol/L) and ‘Alb’ is the serum albumin (g/l).
