# Supplementary material for: Optimization of parathyroid 11C-choline PET protocol for localization of parathyroid adenomas in patients with primary hyperparathyroidism
Source: EJNMMI Res. 2019 Jul 31;9:73. doi: 10.1186/s13550-019-0534-5 (PMC6669228; doi:10.1186/s13550-019-0534-5)
Supplement: Supplementary file 2 — 11C-choline PET/CT (DOCX 16 kb) [file 13550_2019_534_MOESM2_ESM.docx]

**Optimization of parathyroid ^11^C-choline PET protocol for localization of parathyroid adenomas in patients with primary hyperparathyroidism**

Milou E Noltes, Schelto Kruijff, Walter Noordzij, Eef D Telenga, David Vállez García, Malgorzata Trofimiuk-Müldner, Marta Opalińska, Alicja Hubalewska-Dydejczyk, Gert Luurtsema, Rudi AJO Dierckx, Mostafa El Moumni, Ronald Boellaard, Adrienne H Brouwers

**Correspondence to:**

A.H. Brouwers, MD, PhD

Department of Nuclear Medicine and Molecular Imaging

University Medical Center Groningen

[a.h.brouwers@umcg.nl](mailto:a.h.brouwers@umcg.nl)

**Additional file 2: ^11^C-choline PET/CT**

Briefly, ^11^C-choline was produced under GMP-compliant conditions using cassette-based Eckert & Ziegler PharmTracer synthesis module. Radiochemical yield ranged from 28-52% decay corrected and was calculated from ^11^CO_2_. The synthesis time was 25 minutes and ^11^C-choline met the requirements for molar activity. The end product was >95% radiochemically pure, isotonic, and complied with the prospective specification for endotoxins, sterility and organic solvents.

Patients had to fast for six hours while drinking one liter of water, tea and/or coffee without milk and sugar prior to the PET/CT procedure. First, a low dose CT (ldCT) was performed for attenuation correction of the PET images, with 120 kV, Quel ref mAs of 30 and a pitch of 1.5 on a 40 or 64-slice CT (Biograph mCT, Siemens). Scan area involved one bed position, was from the lower jaw to the heart and was recorded in listmode.

All images were iteratively reconstructed using three iterations, 21 subsets with 5 mm FWHM Gaussian filter, including time of flight and resolution modelling.
