# Supplementary material for: Optimization of parathyroid 11C-choline PET protocol for localization of parathyroid adenomas in patients with primary hyperparathyroidism
Source: EJNMMI Res. 2019 Jul 31;9:73. doi: 10.1186/s13550-019-0534-5 (PMC6669228; doi:10.1186/s13550-019-0534-5)
Supplement: Supplementary file 3 — Inter-observer agreement (DOCX 15 kb) [file 13550_2019_534_MOESM3_ESM.docx]

**Optimization of parathyroid ^11^C-choline PET protocol for localization of parathyroid adenomas in patients with primary hyperparathyroidism**

Milou E Noltes, Schelto Kruijff, Walter Noordzij, Eef D Telenga, David Vállez García, Malgorzata Trofimiuk-Müldner, Marta Opalińska, Alicja Hubalewska-Dydejczyk, Gert Luurtsema, Rudi AJO Dierckx, Mostafa El Moumni, Ronald Boellaard, Adrienne H Brouwers

**Correspondence to:**

A.H. Brouwers, MD, PhD

Department of Nuclear Medicine and Molecular Imaging

University Medical Center Groningen

[a.h.brouwers@umcg.nl](mailto:a.h.brouwers@umcg.nl)

**Additional file 3: Inter-observer agreement**

The scans were reviewed by two nuclear medicine physicians from the Department of Endocrinology, Jagiellonian University, Medical College of Krakow in Poland and by a nuclear medicine physician and a resident from the Department of Nuclear Medicine and Molecular Imaging, University Medical Center Groningen of Groningen in The Netherlands. The observers had 3, and 8-10 years of experience in nuclear medicine.

The assessment was carried out using the soft-ware program Syngo.Via VB10B (Siemens) in the Netherlands and Symbia.net (Siemens) in Poland. Observers were blinded to patient and medical information, ratings by other readers and the scan duration. Also, the scans were anonymized with randomly assigned numbers.
