# Supplementary material for: Optimization of parathyroid 11C-choline PET protocol for localization of parathyroid adenomas in patients with primary hyperparathyroidism
Source: EJNMMI Res. 2019 Jul 31;9:73. doi: 10.1186/s13550-019-0534-5 (PMC6669228; doi:10.1186/s13550-019-0534-5)
Supplement: Supplementary file 4 — Results optimal uptake time of tracer (DOCX 125 kb) [file 13550_2019_534_MOESM4_ESM.docx]

**Optimization of parathyroid ^11^C-choline PET protocol for localization of parathyroid adenomas in patients with primary hyperparathyroidism**

Milou E Noltes, Schelto Kruijff, Walter Noordzij, Eef D Telenga, David Vállez García, Malgorzata Trofimiuk-Müldner, Marta Opalińska, Alicja Hubalewska-Dydejczyk, Gert Luurtsema, Rudi AJO Dierckx, Mostafa El Moumni, Ronald Boellaard, Adrienne H Brouwers

**Correspondence to:**

A.H. Brouwers, MD, PhD

Department of Nuclear Medicine and Molecular Imaging

University Medical Center Groningen

[a.h.brouwers@umcg.nl](mailto:a.h.brouwers@umcg.nl)

**Supplementary material 4: results optimal uptake time of tracer**

The ratios adenoma/muscle and adenoma/T1 became constant from the uptake time of 20-30 min p.i. onwards (Fig supplementary material 4). The adenoma/aorta ratio continued to increase over time (Fig supplementary material 4), from 1.70 to 5.76, as activity was cleared from the bloodpool (Table supplementary material 4). In the uptake time of 0-10 versus 10-20 min p.i. the adenoma/aorta ratio increased from 1.70 to 3.75 (p=0.008) and the adenoma/muscle ratio decreased from 3.59 to 2.67 (p=0.008) (Table supplementary material 4). In the uptake time of 10-20 versus 20-30 min there was only a significant difference in adenoma/aorta ratio (p=0.008) with an increase from 3.75 to 5.10, although the adenoma/muscle (decrease from 2.67 to 2.52) ratio is just slightly beyond the level of significance (p=0.021). From the uptake time of 20-30 min p.i. onwards, there were no significant differences with upcoming uptake times for any ratio and p-values were higher compared to previous uptake times (p>0.043).

| **Table supplementary material 4** Descriptive statistics of the adenoma/aorta, adenoma/muscle and adenoma/T1 ratios for the different uptake times in n=9 patients. | | | | | | | | |
| --- | --- | --- | --- | --- | --- | --- | --- | --- |
|  | |  | | **Uptake time of tracer (min)** | | | | |
|  | | **00-10** | **10-20** | | **20-30** | **30-40** | **40-50** | **50-60** |
|  | **Adenoma/aorta** | | | | | | | |
| **Median** | | 1.70 | 3.75 | | 5.10 | 4.49 | 6.02 | 5.76 |
| **SD** | | .76 | 2.13 | | 2.68 | 3.71 | 49.02 | 2.87 |
| **IQ** | | 1.07 | 2.76 | | 3.25 | 3.28 | 4.70 | 4.87 |
|  | **Adenoma/muscle** | | | | | | | |
| **Median** | | 3.59 | 2.67 | | 2.52 | 2.35 | 2.84 | 2.62 |
| **SD** | | 3.38 | 2.61 | | 2.26 | 2.29 | 1.99 | 1.14 |
| **IQ** | | 4.19 | 2.93 | | 2.65 | 2.38 | 2.46 | 2.13 |
|  | **Adenoma/T1** | | | | | | | |
| **Median** | | 2.30 | 1.96 | | 2.09 | 2.07 | 2.55 | 1.80 |
| **SD** | | 1.42 | 1.35 | | 1.28 | 1.34 | 1.59 | .90 |
| **IQ** | | 1.67 | 1.60 | | 1.77 | 1.67 | 1.72 | 1.78 |

*T1 first* thoracic vertebra, *SD* standard deviation, *IQ* interquartile range.

Only 7 and 5 patients were scanned until 50 and 60 minutes postinjection, respectively.


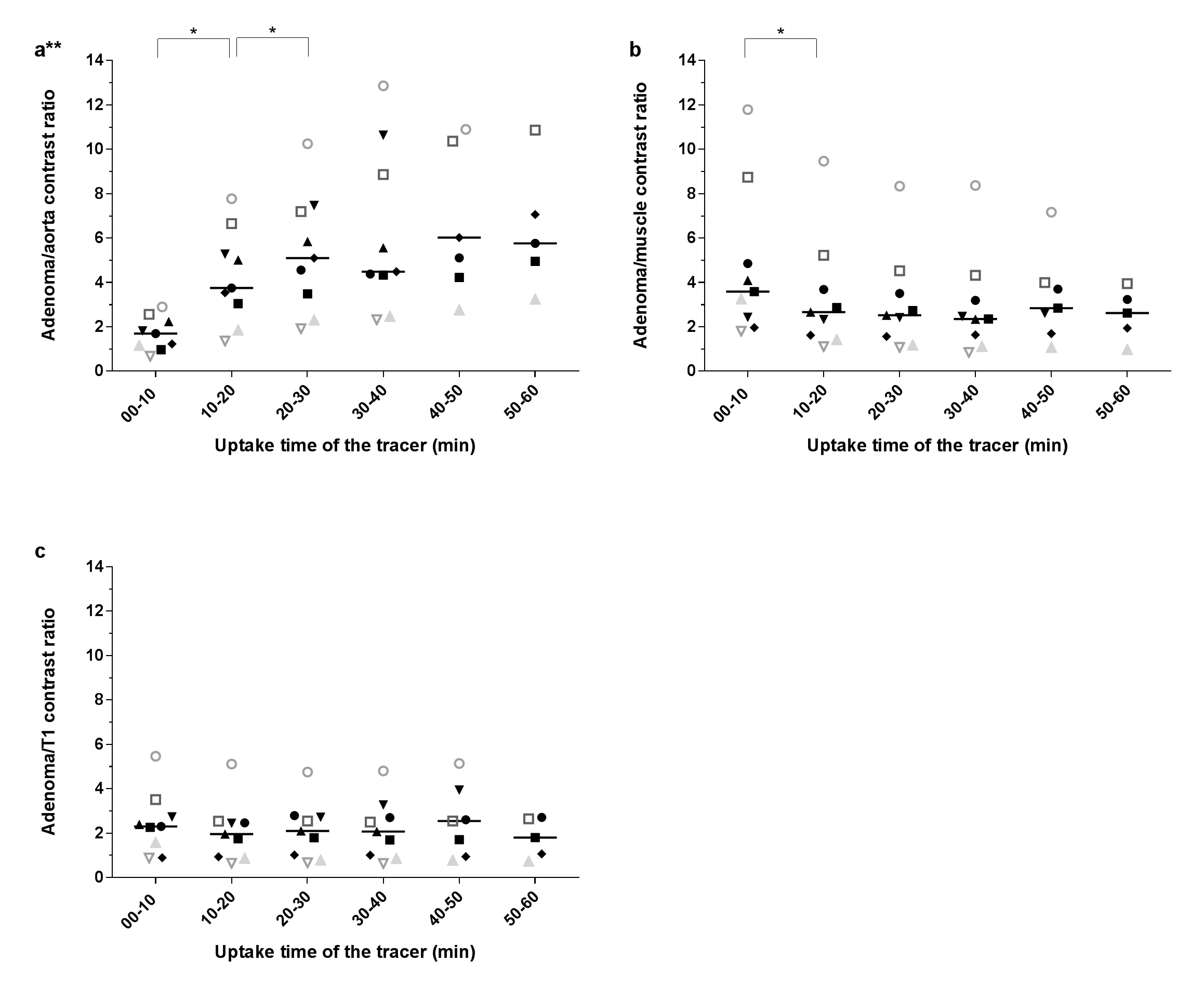
**Fig** **supplementary material 4.** Scatter plot of parathyroid adenoma to background contrast in n=9 patients

Scatter plots with median values of **a**** Adenoma/aorta ratio. **b** Adenoma/muscle ratio. **c** Adenoma/thoracic vertebra T1 ratio. *T1* first thoracic vertebra, *Uptake time of tracer* time (in min) between injection of ^11^C-choline and scanning procedure, ***significant difference (p<0.01), ****one outlier in adenoma/aorta ratio removed at timeframe of 40-50 min postinjection, as the figure was otherwise not readable.

Only 7 and 5 patients were scanned until 50 min and 60 min postinjection, respectively.
