# Supplementary material for: Optimization of parathyroid 11C-choline PET protocol for localization of parathyroid adenomas in patients with primary hyperparathyroidism
Source: EJNMMI Res. 2019 Jul 31;9:73. doi: 10.1186/s13550-019-0534-5 (PMC6669228; doi:10.1186/s13550-019-0534-5)
Supplement: Supplementary file 5 — Results radioactivity to be administered (DOCX 196 kb) [file 13550_2019_534_MOESM5_ESM.docx]

**Optimization of parathyroid ^11^C-choline PET protocol for localization of parathyroid adenomas in patients with primary hyperparathyroidism**

Milou E Noltes, Schelto Kruijff, Walter Noordzij, Eef D Telenga, David Vállez García, Malgorzata Trofimiuk-Müldner, Marta Opalińska, Alicja Hubalewska-Dydejczyk, Gert Luurtsema, Rudi AJO Dierckx, Mostafa El Moumni, Ronald Boellaard, Adrienne H Brouwers

**Correspondence to:**

A.H. Brouwers, MD, PhD

Department of Nuclear Medicine and Molecular Imaging

University Medical Center Groningen

[a.h.brouwers@umcg.nl](mailto:a.h.brouwers@umcg.nl)

**Additional file 5: results radioactivity to be administered**

There was no significant difference in SUV_mean_ for aorta, muscle and T1 between the different scan durations (p>0.123). The SUV_max_ for aorta in the scan duration of 1, 2.5 and 5 minutes all differed statistically from the 10 min scan duration (all p=0.012). There was only a significant difference in SUV_max_ for muscle between a 1 min versus 10 min scan duration (p=0.012). The SUV_max_ for T1 was not significant different between the different scan durations (p>0.017).

| **Table additional file 5.** Descriptive statistics of the average SUVs for background tissues (aorta, muscle and first thoracic vertebra (T1)) for the different scan durations in n=8 patients. | | | | | | | | | | | | |  |
| --- | --- | --- | --- | --- | --- | --- | --- | --- | --- | --- | --- | --- | --- |
| **SUV_mean_ for aorta** | | | | | | | **SUV_max_ for aorta** | | | | | |  |
| **Scan duration (min)** | | | | | |  | **Scan duration (min)** | | | | |  |  |
|  | **1** | | **2.5** | | **5** | **10** |  | | **1** | **2.5** | **5** | **10** |  |
| **Median** | .92 | | .91 | | .91 | .87 | **Median** | | 2.14 | 1.58 | 1.38 | 1.22 |  |
| **SD** | .23 | | .22 | | .22 | .22 | **SD** | | .65 | .36 | .36 | .30 |  |
| **IQ** | .39 | | .37 | | .37 | .33 | **IQ** | | 1.21 | .63 | .65 | .53 |  |
| **SUV_mean_ for muscle** | | | | | | | | **SUV_max_ for muscle** | | | | | |
| **Scan duration (min)** | | | | | | | | **Scan duration (min)** | | | | | |
|  | **1** | **2.5** | | **5** | | **10** |  | | **1** | **2.5** | **5** | **10** |  |
| **Median** | 1.57 | 1.57 | | 1.57 | | 1.59 | **Median** | | 2.16 | 1.97 | 1.90 | 1.83 |  |
| **SD** | .33 | .33 | | .33 | | .31 | **SD** | | .36 | .37 | .31 | .31 |  |
| **IQ** | .57 | .57 | | .58 | | .53 | **IQ** | | .75 | .70 | .60 | .64 |  |
| **SUV_mean_ for T1** | | | | | | | | **SUV_max_ for T1** | | | | | |
| **Scan duration (min)** | | | | | | | | **Scan duration (min)** | | | | | |
|  | **1** | **2.5** | | **5** | | **10** |  | | **1** | **2.5** | **5** | **10** |  |
| **Median** | 2.08 | 2.07 | | 2.07 | | 2.06 | **Median** | | 2.99 | 2.90 | 2.87 | 2.81 |  |
| **SD** | .54 | .55 | | .54 | | .52 | **SD** | | .68 | .63 | .62 | .55 |  |
| **IQ** | .90 | .91 | | .90 | | .79 | **IQ** | | 1.14 | 1.01 | .92 | .81 |  |

*SD* standard deviation, *IQ* interquartile range, *SUV* standardized uptake value


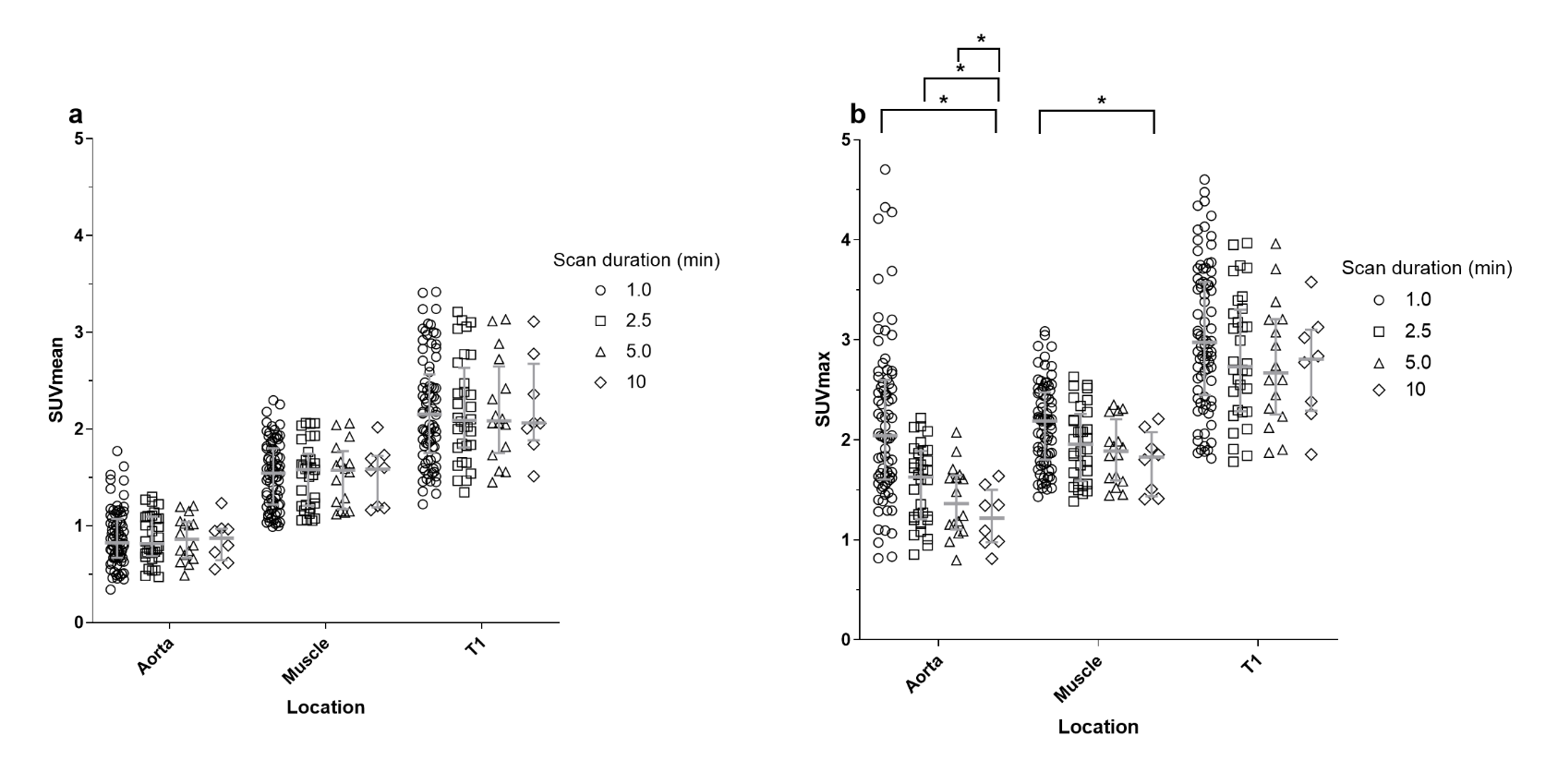
**Fig. additional file 5.** Scatter plot of SUV_mean_ and SUV_max_ of background tissues (aorta, muscle and first thoracic vertebra (T1)) in the different scan durations (1, 2.5, 5 and 10 min) in n=8 patients.

Scatter plot representing median values and interquartile ranges of **a** SUV_mean_ for background tissue in the different scan durations. **b** SUV_max_ for background tissue in the different scan durations. *SUV* standardized uptake value, *T1* first thoracic vertebra , *** significant difference (p<0.017)
